# *GSTM1* null and *GSTT1* null: predictors of cisplatin-caused acute ototoxicity measured by DPOAEs

**DOI:** 10.1007/s00109-020-01921-y

**Published:** 2020-05-20

**Authors:** Barna Budai, Péter Prekopp, László Noszek, Erika R. Kovács, Márta Szőnyi, Dániel J. Erdélyi, Krisztina Bíró, Lajos Géczi

**Affiliations:** 1 Department of Molecular Genetics, National Institute of Oncology, Rath Gy. u. 7-9, Budapest, 1122 Hungary; 2 Department of Otorhinolaryngology, Head and Neck Surgery, Semmelweis University, Budapest, Hungary; 3 ENT Department, Szent Imre Hospital, Budapest, Hungary; 4 2nd Department of Pediatrics, Semmelweis University, Budapest, Hungary; 5 Department of Oncology, “Szent Margit” Hospital, Budapest, Hungary; 6 Department of Medical Oncology and Clinical Pharmacology “C”, National Institute of Oncology, Budapest, Hungary

**Keywords:** Acute ototoxicity, Cisplatin, DPOAEs, GST polymorphisms, Testicular cancer

## Abstract

**Abstract:**

Preventing the ototoxicity caused by cisplatin is a major issue yet to be overcome. Useful preventive treatments will soon be available. Consequently, the next step is to filter out those patients who are more prone to develop ototoxicity. The aim of this study was to prospectively evaluate potential predictive markers of acute ototoxicity as determined by measures of distortion product otoacoustic emissions (DPOAEs). A total of 118 patients from our previous DPOAE analysis were put under evaluation. Ototoxic cases were divided according to unilateral (*n* = 45) or bilateral (*n* = 23) involvement. The clinicopathological characteristics, hearing test results, germline *GSTT1*, *GSTM1*, and *GSTP1* polymorphisms, and common laboratory parameters were included in the new analysis. Univariate and multivariate statistical tests were applied. According to multivariate logistic regression, the only independent predictor of unilateral ototoxicity (vs. non-affected) was a *GSTM1* null genotype (OR = 4.52; 95%CI = 1.3–16.3), while for bilateral damage, the *GSTT1* null genotype (OR = 4.76; 1.4–16) was a predictor. The higher starting serum urea level was characteristic of bilateral ototoxicity; however, the only independent marker of bilateral (vs. unilateral) ototoxicity was the presence of *GSTT1* null genotype (OR = 2.44; 1.23–4.85). Different processes, involving the *GSTM1* and *GSTT1* genotypes, respectively, govern the development of acute unilateral and bilateral ototoxicities. Further research is needed to clarify these processes. Based on the above findings, patients whom are at risk may be selected for otoprotective therapies.

**Key messages:**

- The acute ototoxicity was determined by DPOAE in 118 testicular cancer patients.
- *GSTM1* null was the only marker of unilateral ototoxicity (vs. non-affected).
- The only marker of bilateral hearing loss (vs. non-affected) was the *GSTT1* null.
- *GSTT1* null was also the marker of bilateral vs. unilateral ototoxicity.
- A high-risk group may be selected for new, individualized otoprotective treatment.

## Introduction

Germ cell tumors are among the most frequent neoplasms identified in men from 15–44 years of age [1], even at an advanced stage germ cell tumors can be successfully treated by the use of a combination of chemotherapeutical regimens [2]. In the past decades, the 5-year tumor-free survival rate gradually improved and now it exceeds 90%. One of the most important components of the combined chemotherapeutic regimen is cisplatin with its most frequently appearing side effect, ototoxicity [3]. One of the major requirement from a chemotherapeutic drug is to assure the long-term survival of patients, which can be achieved by applying the potentially ototoxic platinum compounds. In clinical practice, ototoxicity is the major dose-limiting side effect of cisplatin treatments [4]. The reported incidence of cisplatin ototoxicity varies from 9 to 91%, based on the differences in chemotherapeutical regimens, patients population, and the definition of ototoxicity, along with variations and inconsistencies in the assessment and grading of hearing loss [5]. Preventing ototoxicity is crucial; however currently available methods to avoid ototoxic side effects are limited, even in the case of endangered patients: use of platinum drugs with less ototoxic potential (usually carboplatin) [4], reduced dose of cisplatin [6], use of otoprotective drugs [7–10], or advices to avoid concomitant or further noise injuries [3], etc. Many ongoing clinical trials are addressing this issue suggests that useful preventive treatments will soon be available [4]. Consequently, the next step is to identify those patients, who are going to develop ototoxicity; however, predicting which patients will experience ototoxicity is a significant clinical challenge. The risk of developing hearing loss from drugs is most often correlated with dosage, but this correlation is highly variable. Individual susceptibility to hearing damage is influenced by multiple biochemical, physiologic, and genetic factors [11]. By using results from international literatures [12–14], it can be concluded that the appearance of ototoxic effect of platinum-containing chemotherapeutic drug is influenced by the presence or absence of certain types of glutathione-S-transferase (GST) enzymes, which are partly responsible for cisplatin metabolism. These earlier experiments were retrospectively conducted and subjective methods using audiometers were utilized for the audiological measurements.

The aim of this study was to prospectively investigate for possible predictive markers of acute ototoxicity, which was determined by using distortion product otoacoustic emission (DPOAE) measurements. The study evaluated the modifications in the acute phase immediately after the 1st cisplatin cycle; thus, the findings do not address changes in DPOAEs due to chronicity or permanent damage to the cochlea. Besides *GSTT1*, *GSTM1*, and *GSTP1* polymorphisms, other laboratory parameters and past records of hearing tests were examined. The a priori identification of a high-risk group can served the basis for a better definition of individualized treatment and the targeted use of new otoprotective drugs.

## Experimental details

### Patients

A total of 118 patients with testicular cancer were treated with combination of cisplatin + bleomycin + etoposide were considered for the investigation of predictive markers for acute ototoxicity. These patients represent a subcohort (i.e., those without missing laboratory data) of patients presented in our previous study [15].

The Institutional Ethical Committee and the Hungarian Medical Research Council approved the study (323-101/2005-1018EKU). All patients signed an informed consent.

### Hearing evaluation and identification of ototoxicity

At the treatment site, patient’s clinical records were reviewed for the following factors: past hearing complaints, present hearing complaints, noise injuries, exposure to noise pollution, hearing loss risk factors, and smoking habits. The historical data were also considered in the analysis. The description of DPOAE measurement and the exclusion criteria for patients including presbyacusis, abnormal tympanic cavity pressure, etc. were detailed in our previous study [15]. Ototoxicity was established by measuring DPOAEs separately for both ears before and immediately after the 1st cycle of cisplatin-based chemotherapy at 100 mg/m^2^/5 days.

**Fig. 1:**
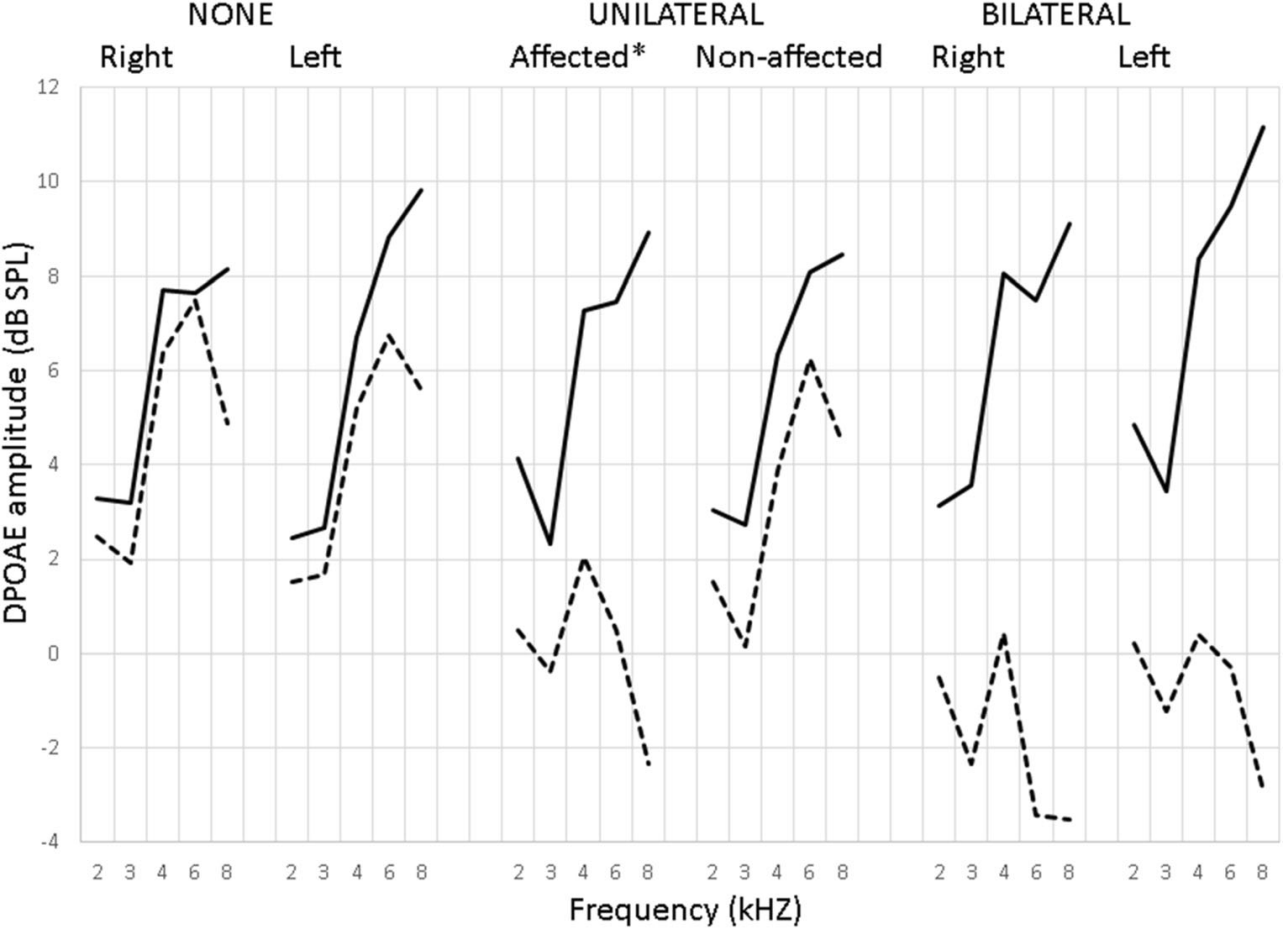
Results of DPOAE measurement according to the number of affected ears. The values are expressed as mean DPOAE before (solid line) and after (dotted line) the 1st cisplatin cycle. *In 23 patients the right ear and in 22 patients the left ear was affected

The ototoxic effect of cisplatin is known to primarily occur at higher frequencies [16]; therefore, to correctly select the affected patients, only the frequencies 2, 3, 4, 6, and 8 kHz were considered. Dreisbach et al. [17] concluded that “overall, a 4-9 dB change in DPOAE level is considered statistically significant for short, long term monitoring of DPOAEs at frequencies lower than 8 kHz.” According to Reavis et al. [18], the standard error of DPOAE measurement (SEM) was defined by test-retest changes, and the 90% DPOAE shift reference intervals were calculated for each frequency. The reference intervals were as follows: ± 5.47 dB at 2 kHz; ± 5.81 dB at 3 kHz; ± 6.14 dB at 4 kHz; ± 6.92 dB at 6 kHz; and ± 5.83 dB at 8 kHz. Based on the above statement and the calculated reference intervals, we defined acute ototoxicity at a ≥ 7 dB decrease in DPOAE amplitudes. Moreover, patients were considered unilaterally or bilaterally affected. Out of 118 patients, 23 presented with bilateral and 45 with unilateral ototoxicity (Fig. 1). In both groups, 67% of patients showed significant changes in at least one of the two highest frequencies. In 46% of affected patients, a significant DPOAE shift was detected in two or more frequencies in at least one ear.

### Clinicopathological parameters

In addition to patient’s age, histology result (seminoma vs. non-seminoma), disease stage (1 vs. 2–3), and blood pressure (normotension vs. hypertension) at the beginning of treatment were used in the analysis. The following laboratory data were recorded before the treatment were used for the analysis: white blood cell and platelet count, serum level of hemoglobin and hematocrit, and levels of aspartate aminotransferase, alanine transaminase, gamma-glutamyl transferase, urea, creatinine, glucose, bilirubin, Na, K, Ca, and Mg. At the end of the first treatment cycle, the white blood cell and platelet count; serum level of hemoglobin, hematocrit, and urea; and creatinine levels were available for investigation.

### Genotyping

Blood samples were taken from patients and DNA prepared as described earlier [19]. *GSTT1* and *GSTM1* null genotypes and the *GSTP1* Ile105Val (rs1695) polymorphism were evaluated by multiplex PCR and the PCR-RFLP method, respectively, as described in detail by Kiran et al [20].

### Statistical analyses

All continuous and categorical data were compared in the three subgroups with one-way ANOVA (or Kruskal-Wallis) and chi-squared (or exact) tests, respectively. Post hoc tests were applied to find all pairwise differences between the subgroups. In the case of parameters available before and after treatment, the changes were also analyzed. *P* < 0.05 value was considered statistically significant. A series of logistic regressions were applied to find independent markers of unilateral and bilateral, or any ototoxicity, and, moreover, to differentiate between unilateral and bilateral ototoxicities. All variables, with *P* < 0.1 of post hoc tests in univariate analysis, were introduced in the respective logistic regression analysis to determine the independent markers of acute ototoxicity. The Hardy-Weinberg equilibrium was tested for *GSTP1* polymorphism on www.dr-petrek.eu/documents/HWE.xls. The NCSS program (NCSS 12 Statistical Software (2018). NCSS, LLC. Kaysville, Utah, USA, ncss.com/software/ncss.) was used for the statistical analyses.

## Results

The distribution of the clinicopathological parameters of the patients are presented in Table 1.

**Table 1:** Hearing test results and genomic and laboratory parameters of the patients according to ototoxicity (none, unilateral, or bilateral) measured by DPOAEs

Parameters	Patients presenting ototoxicity		
	None (*N* = 50)	Unilateral (*N* = 45)	Bilateral (*N* = 23)
	Median (95% CI)	Median (95% CI)	Median (95% CI)
Age (range) [years]	31.5 (29 to 36)	34 (32 to 36)	33 (28 to 40)
Histology seminoma/non-seminoma	13/37	12/33	3/20
Stage 1/2–3	17/33	20/25	12/11
Former hearing complaints n/y/NA	36/2/12	27/5/13	11/2/10
Noise injury n/y/NA	24/14/12	19/13/13	6/7/10
Noise pollution n/y/NA	23/15/12	19/13/13	6/7/10
Hearing loss risk factors n/y/NA	30/9/11	20/14/11	7/7/9
Hearing complaints before the 1st cycle n/y/NA	36/0/14	30/1/14	12/0/11
Hearing complaints after the 1st cycle n/y/NA	31/6/13	26/5/14	11/2/10
Smoking ever n/y/NA	13/25/12	13/19/13	2/11/10
Hypertension before the 1st cycle n/y/NA	31/9/10	18/11/16	10/5/8
*GSTT1* wild type/null	40/10	40/5	13/10*
*GSTM1* wild type/null	31/19	18/27**	13/10
*GSTP1* wild type/hetero/homo	21/25/4	22/19/4	11/12/0
White blood cell count before the 1st cycle [G/L]	7.5 (6.5 to 8.4)	7.5 (6.8 to 8.5)	7.3 (6.5 to 9.1)
White blood cell count after the 1st cycle [G/L]	8.5 (7.7 to 9.5)	8.7 (8.1 to 9.6)	8.1 (7.1 to 8.7)
White blood cell count change [% of before]	6 (− 5 to 12.7)	13.3 (2.5 to 26.7)	8.1 (− 3.9 to 23.2)
Hemoglobin before the 1st cycle [mmol/L]	14.8 (14.4 to 15.1)	15.1 (14.6 to 15.4)	14.7 (13.9 to 15.5)
Hemoglobin after the 1st cycle [mmol/L]	13.7 (13.1 to 14)	13.9 (13.5 to 14.2)	13.6 (12.8 to 14.3)
Hemoglobin change [% of before]	− 6.7 (− 8.9 to − 4.9)	− 7.7 (− 9.4 to − 4.9)	− 6.5 (− 10.6 to − 3.5)
Hematocrit before the 1st cycle [L/L]	0.44 (0.44 to 0.46)	0.45 (0.44 to 0.47)	0.45 (0.43 to 0.47)
Hematocrit after the 1st cycle [L/L]	0.41 (0.4 to 0.42)	0.41 (0.4 to 0.42)	0.41 (0.38 to 0.42)
Hematocrit change [% of before]	− 7.3 (− 10.9 to − 5.4)	− 8.6 (− 10.6 to − 6.5)	− 8.8 (− 12.8 to − 4.9)
Platelet count before the 1st cycle [G/L]	254 (240 to 275)	249 (229 to 266)	247 (225 to 297)
Platelet count after the 1st cycle [G/L]	268 (234 to 296)	251 (219 to 270)	221 (208 to 282)
Platelet count change [% of before]	− 3.1 (− 38 to 4.9)	0 (− 2.6 to 4.7)	− 2.7 (− 9 to 2.5)
Creatinine before the 1st cycle [μmol/L]	89 (84 to 94)	89 (81 to 95)	89 (85 to 94)
Creatinine after the1st cycle [μmol/L]	89 (78 to 92)	82 (77 to 89)	87 (79 to 98)
Creatinine change [% of before]	− 1.8 (− 10.1 to 2.5)	− 3.2 (− 12.9 to 0)	− 5.7 (− 11.7 to 1.4)
Urea before the 1st cycle [mmol/L]	4.2 (3.9 to 4.9)	4.6 (4.2 to 5)	5.2 (4.8 to 5.9)***
Urea after the 1st cycle [mmol/L]	5 (4.6 to 5.3)	5.6 (4.9 to 6.2)	4.9 (4.6 to 6.3)
Urea change [% of before]	12.2 (0 to 28.6)	15.5 (7.1 to 35.4)	− 1.7 (− 12.9 to 15)****
Aspartate aminotransferase [IU/L]	23 (21 to 27)	24 (23 to 26)	23 (21 to 26)
Alanine transaminase [IU/L]	26.5 (20 to 30)	28 (25 to 33)	25 (20 to 31)
Gamma-glutamyl transferase[IU/L]	33.5 (25 to 40)	41 (34 to 47)	33 (27 to 40)
Glucose [mmol/L]	5.2 (4.9 to 5.4)	5.1 (5 to 5.3)	5.2 (5.1 to 5.8)
Bilirubin [μmol/L]	11.2 (8.5 to 13.5)	11.3 (9.4 to 14)	10.6 (8 to 16.2)
Na [mmol/L]	138 (137 to 138)	138 (137 to 139)	139 (138 to 140)
K [mmol/L]	4.5 (4.3 to 4.6)	4.4 (4.3 to 4.6)	4.3 (4.2 to 4.5)
Ca [mmol/L]	2.5 (2.45 to 2.54)	2.5 (2.46 to 2.52)	2.5 (2.49 to 2.55)
Mg [mmol/L]	0.85 (0.83 to 0.89)	0.84 (0.82 to 0.87)	0.85 (0.8 to 0.87)

*CI* confidence interval, *hetero* heterozygous, *homo* homozygous mutant, *n* no, *NA* not available, *y* yes

^*^P = 0.008 (0 vs. 2 *P* = 0.037; 1 vs. 2 *P* = 0.002)

^**^0 vs. 1 *P* = 0.032

^***^P = 0.034 (0 vs. 2 *P* = 0.02; 1 vs. 2 *P* = 0.023)

^****^P = 0.031 (1 vs. 2 *P* = 0.007)

In the case of the *GSTP1*, there was no deviation from the Hardy-Weinberg equilibrium (*P* = 0.195). The frequency of the null genotypes of *GSTT1* and *GSTM1* was 21.2% and 47.5%, respectively, (see Table 1).

There was a statistically significant accumulation of patients (43%) with the *GSTT1* null genotype presenting with bilateral ototoxicity (Table 1), while the distribution of the null genotype was lower for unaffected or unilaterally affected patients (20% and 11%, respectively). The presence of the *GSTM1* null genotype was significantly more frequent in patients with only one ear involvement (60%), while this value was 38–43% for the other subgroups.

In comparison with the pre-treatment measures, white blood cell count and urea level significantly increased at the end of first cycle, while the hemoglobin, hematocrit, and creatinine levels were decreased (Wilcoxon signed-rank test) in all subgroups, except for the white blood cell count and the urea level in patients with bilateral ototoxicity.

The initial level of urea was higher in patients with acute bilateral ototoxicity than in the other two subgroups (*P* = 0.02 and *P* = 0.023). Interestingly, this high initial level of serum urea remained unchanged even after the 1st cycle of chemotherapy, while in the other subgroups, a ~ 20% increase was observed.

The following variables were introduced in the logistic regression model of 0 vs. 1 involved ear: *GSTM1*, “urea after,” and aspartate aminotransferase. Only the presence of the *GSTM1* null proved to be an independent marker of unilateral ototoxicity with OR = 3.62; (95% CI 1.3–10.5); *P* = 0.018. The area under the receiver operating curve (ROC) was 0.624.

The *GSTT1*, glucose, and urea levels were used as covariates in the logistic regression model for 0 vs. two affected ears. Only the *GSTT1* null was an independent marker of bilateral ototoxicity: OR = 3.6; (1.1–11.4); *P* = 0.029. The area under the ROC curve was 0.617.

The model for unilateral vs. bilateral involvement included the following parameters: the *GSTT1*, urea, glucose, and “white blood cell count after,” while “urea change” was excluded because of a multicollinearity. Only the *GSTT1* null was found to be an independent predictor of acute bilateral ototoxicity compared with unilateral damage: OR = 4.31; (2.8–4.3); *P* = 0.033. The area under the ROC curve was 0.706.

If ototoxicity (either or both ears involved vs. non-affected patients) was considered and age and *GSTM1* were used as covariates, logistic regression resulted in no significant variables.

## Discussion

In this study, we aimed to evaluate the potential predictive markers of cisplatin-caused acute ototoxicity as determined by DPOAEs to give further assistance to oncologists in designing individual therapies for patients at risk. DPOAE measures indicated earlier damage of outer hair cells compared with the outcomes of routine audiometric tests [21]. In our previous study [15], acute ototoxicity was demonstrated by measuring DPOAEs before and after the 1st cycle of a cisplatin-based treatment in patients with testicular cancer. In the present report, we divided patients into three subgroups according to the nature of ototoxicity, i.e., no ototoxicity, unilateral toxicity, and bilateral ototoxicity. Data regarding hearing complaints, routine laboratory parameters, and three germline genetic polymorphisms (*GSTM1*, *GSTT1*, and *GSTP1*) were investigated. The frequencies of *GSTM1* null and *GSTT1* null genotypes were similar to those published for a Hungarian population (47.4% and 20.6%, respectively) [22]. The distributions of *GSTP1* genotypes were in Hardy-Weinberg equilibrium. We have observed that a *GSTM1* null was associated with unilateral involvement, while the *GSTT1* null was associated with a bilateral acute ototoxicity. It is likely that other non-investigated parameters can also contribute to the development of an ototoxicity, because the observed areas under the ROC curves for both markers were ~ 0.6.

There are few reports, which have studied *GST* polymorphisms in cisplatin-induced acute toxicity (see Table 2). To our knowledge, this is the first study investigating the role of *GSTM1, GSTT1*, and *GSTP1* polymorphisms in acute ototoxicity after the 1st cycle of cisplatin determined by DPOAEs in patients with testicular cancer. Khrunin et al. did not find any association between the *GSTM1*, *GSTT1*, or *GSTP1* gene variants, and ototoxicity measured audiometrically after the 2nd cycle of cisplatin [23]. Talach et al. and Jurajda et al. measured ototoxicity by pure-tone audiometry and found significant associations with the *GSTT1* +/+ genotype both after the 1st and 2nd cisplatin cycles [12, 24]. Barahmani et al. also investigated *GST* polymorphisms in patients presenting with a grade 3 audiologic toxicity during cisplatin treatment, but there was no correlation between development or time to the development of ototoxicity and the *GST* polymorphisms [25]. After presenting these results, we can discuss those studies, which investigated the association between *GST* gene variants and late ototoxicity. Unfortunately, there are no investigations, which examined the association of these polymorphisms and the late-onset ototoxicity measured by DPOAEs. Olgun et al. [26] reported the use of DPOAEs; but in addition to pure-tone audiometry, unfortunately, the detailed results of DPOAEs were not reported and ototoxicity was considered based only on the audiogram. Peters et al. considered all cycles of cisplatin to define ototoxicity in 39 patients. However, even though the pure-tone audiometry was performed after each cycle, the rate of ototoxic cases was not reported separately after each treatment cycle [31]. Aside from a consistently bilateral involvement, no association was reported for *GSTM1*, *GSTT1*, and *GSTP1* polymorphisms. Similarly, Ross et al. [27] and Khrunin et al. [23] did not find any significant association between the above gene variants and late-onset ototoxicity. Among others, the significantly higher rate (69% vs 46% in non-affected patients) of concomitant treatments with ototoxic side effects (aminoglycosides and cranial irradiation) of patients with hearing loss [27], or patients’ characteristics (women, median 52 years, ovarian cancer), and that cisplatin treatment was stopped if a grade > 1 of ototoxicity occurred [23] may mask the effect of *GST* polymorphisms. Talach et al. measured ototoxicity by pure-tone audiometry at the end of cisplatin treatment and found no association with any of the above polymorphisms [12]. Interestingly, before and immediately after the 2nd cycle and before the 3rd cycle, they observed a significantly higher rate of ototoxic cases in patients with a wild type *GSTT1* (+/+) genotype. If patients with at least one wild type allele were considered (as in our study), the association was not present. Unfortunately, unilateral and bilateral ototoxicites were not assessed.

**Table 2:** Association between ototoxicity and studied *GST* polymorphisms

Study	*n*	Ototoxicity	Cumulative dose mg/m^2^ cisplatin	Method	*GSTT1* null/null vs others	*GSTM1* null/null vs others	*GSTP1* w/h/m
Present	118	Early	100	DPOAE	OR = 4.1; *p* = 0.004^a + b^ (OR = 3.6; *p* = 0.029)^a^**	OR = 2.4; *p* = 0.032^b^ (OR = 3.6; *p* = 0.018)^b^**	NS
[23]	104	Early	200	PTA	NS	NS	NS
[12, 24]	54	Early	100	PTA	(OR = 6.4; *p* = 0.009)*	NS	NS
[12, 24]	38	Early	200	PTA	(OR = 6.3; *p* = 0.027)*	NS	NS
[25]	34	Any during CHT	75–600	PTA	NS	NS	–
[26]	72	Late	Median ~ 400	DPOAE+ PTA/ABR	–	–	(m vs others OR > 1; *p* = 0.03) (m vs others NS)**
[14]	173	Late	Median ~ 400	PTA	NS	NS (OR = 0.4; *p* = 0.022)**	*p* = 0.021 (m vs others OR = 0.2; *p* < 0.001)**
[27]	162	Late	Median 400	PTA	–	NS	NS
[28]	106	Late	Median 400	PTA	NS (OR = 3.5; *p* = 0.038)**	NS	*p* = 0.046 (w vs others OR = 3.8; *p* = 0.012)**
[23]	104	Late	600	PTA	NS	NS	NS
[29] [13]	90 68	Late Late	Median 265 Median 526	PTA PTA	OR = 0.2; *p* = 0.01 OR = 0.2; *p* = 0.023 (OR = 0.1; *p* = 0.002)**	NS NS	NS –
[30]	42	Late	Mean 635	PTA	–	–	(w vs others NS, but at ≤ 4 kHz OR = 0.1; *p* = 0.02)
[31]	39	Late	Mean 236	PTA	NS	NS	NS
[12]	37	Late	600	PTA	NS (NS)*	–	–
[32]	238	Late	Median 397	Self-reported	NS	OR = 0.6; *p* = 0.025	(m vs others OR = 0.3; *p* = 0.008)**
[33]	69	Late	Median 300	Hearing aid	–	–	NS (w vs others OR = 0.3; *p* = 0.021)

*ABR* auditory brainstem response, *CHT* chemotherapy, *DPOAE* distorsion product otoacoustic emission, *h* heterozygous genotype, *m* homozygous mutant genotype, *NS* non-significant, *PTA* pure-tone audiometry, *OR* odds ratio, *w* homozygous wild genotype

*+/+ vs others

**Multivariant analysis

^a^Bilateral

^b^Unilateral

Based on our previous [34, 35] and present experiences and on reports from the literature [36–38], we presume that molecular processes that lead to acute or permanent (late) ototoxicity caused by cisplatin can differ. Therefore, comparison of results from studies on acute and chronic ototoxicity should be avoided as this may account for contradictory results in the literature. For example, Oldenburg et al. investigated hearing impairment after > 4 years from the start of cisplatin treatment; thus, their findings [32] are not comparable with our results on acute ototoxicity. The contradiction between the results (*GSTM1* or *GSTT1* null genotype as a protector or facilitator for ototoxicity) relies on different physiological phenomena: early acute ototoxicity may be associated to damage of stria vascularis and supporting cells within the cochlea that have the potential to recover, whereas the damage to outer hair cells results in a permanent hearing loss [37]. In some studies, ototoxicity was only assessed at the completion of chemotherapeutic treatment. Ototoxicity at that stage cannot be compared with acute ototoxicity, because the cumulated cisplatin doses (4–6 × higher than those for acute events) are similar to those presented for chronic events. The fact of incomparability was mirrored by the findings of Choeyprasert et al. who measured ototoxicity by audiometry, just at the end of therapy, and the *GSTT1* null proved to be protective for hearing impairment [13].

The difference between acute and late ototoxicity was also presented in studies by Talach et al. [12] and Jurajda et al. [24] where the early observed significant association of the GSTT1 genotype with ototoxicity disappeared at the end of treatment. According to the data of Table 2, the association between late ototoxicity and the *GSTP1* polymorphism is very controversial. Both the homozygous mutant [14, 26, 32] and the wild genotype [28, 30, 33] were associated with ototoxicity [26, 28] or a protective effect [14, 30, 32, 33], or no effect was found [23, 27, 29, 31].

Interestingly, the *GSTT1* null was found to be associated with hearing damage (presbycusis) at high frequencies, which was determined by audiometry in 50 adults [39], while the presence of the *GSTM1* null genotype was associated with noise-induced hearing loss as determined by pure-tone audiometry in 889 Chinese workers [40]. The latter result led us to hypothesize that our patients with the *GSTM1* null genotype were also prone to noise-induced hearing loss, but a lower noise exposure threshold may be present, because of cisplatin treatment. This presumption should be investigated in further studies.

Regarding the high initial urea levels, acute dehydration can be excluded because the hemoglobin, hematocrit, serum sodium (Na^+^), and potassium (K^+^) levels of patients with further bilateral involvement were within the reference ranges (i.e., they were in the middle of the respective normal intervals). Impaired kidney function can also be excluded because the creatinine and the estimated glomerular filtration rate levels (calculated to check the normal kidney function; data not shown) did not differ from those of other patients. Also, drugs (steroids, cytokines, tetracyclines, etc.), which may increase the urea levels of patients, were not initiated before chemotherapy.

Other factors, such as a high protein diet, presence of hidden infection, inflammation or stress, or other cause(s), may raise the urea levels [41]. In order to verify if a systemic inflammation was present or not, we calculated the systemic immune-inflammation index (SII) (as the C-reactive protein was not available for our patients). There was no significant difference between patients with high vs. low urea level (data not shown), which suggested that high urea levels were not caused by systemic inflammation, or the SII was not a good marker for indicating cancer-caused inflammation in this patient group. Moreover, the yet unidentified urea-increasing processes overcome the well-known urea-increasing effect of cisplatin, which as a later process was observed in our patients. Further investigations should be conducted to reveal the nature of competing processes. On the other hand, a severe (bilateral) ototoxicity may be attributed to the additively enhanced reactive oxygen species formation due to existing high urea levels [42] and cisplatin treatment [3]. None of the studied polymorphisms have been associated with urea level; however, our data shows that elevated level of urea at the initiation of therapy in patients, who later developed severe ototoxicity needs further prospective studies.

There are some limitations of this study. We have not investigated the combination of polymorphisms, as did other authors [14, 25], because the number of cases in the study groups would not allow enough statistical power for drawing such conclusions. There are several common laboratory parameters and markers related to ototoxicity (e.g., [27, 43]), which were not evaluated in our study, since these parameters were not available for all patients. On the other hand, the statistical power was strong in our analysis.

The role of the studied (and not evaluated in our current work) polymorphisms in predicting cisplatin-caused early ototoxicity is far from being clarified. In the future, the most important task would be to find a key to differentiate between those early ototoxic cases, which will recover, and those who are going to develop a permanent hearing impairment. It needs to be emphasized that early ototoxicity is prone to recovery in some patients [37]. In contrast, other patients presenting with early ototoxicity will develop chronic hearing loss in spite of a dose reduction or changing to other platinum-containing drug. Based on our present results, we may hypothesize that there may be several and different competing molecular mechanisms involving GST and supposedly other reactive oxygen species scavenger enzymes [3], for the development of unilateral and bilateral ototoxicities.

For the association of genotypes with unilateral and bilateral ototoxicity, we have only hypothetical explanations. The asymmetry and the genetic difference of the bilateral organs are also well-known; thus, an association of a genotype with unilateral ototoxicity is possible. Another option may be related to the unilateral noise–induced effect during treatment, which may result in hearing damage, because the ears during treatment are more sensitive to intense noise. However, this sensitivity may be related to some gene variants. Last, but not least, it is hypothesized that two molecular mechanisms with different speeds may cause ototoxicity. Due to the asymmetry of organs (which includes the asymmetry in the expression of enzymes), the slow toxicity becomes first unilateral and then bilateral. Our patients were also tested more than 1 year after treatment. According to our as yet unpublished preliminary results, in cases where the acute unilateral ototoxicity became bilateral, 50% of patients had *GSTM1* null and 0% had *GSTT1* null genotypes. So this could be a GSTM1-dependent slow process. The rapid response is characteristic of *GSTT1* null patients, who presented acute and also late (persistent) bilateral ototoxicity: with 67% of them being *GSTT1* null. The situation is further complicated or explained by the fact that improvement is only seen in 12% of patients and only in unilateral cases, who exhibited 0% *GSTT1* nulls and 67% *GSTM1* nulls. Thus, the slow process involves also reversible ototoxicity, and perhaps this is why it seems slow in the development of bilateral damage. Further research is needed to clarify these phenomena.

In conclusion, *GSTM1* null and *GSTT1* null genotypes proved to be independent markers of unilateral and bilateral acute ototoxicities, respectively. The a priori identification of a high-risk group can serve as a basis for a better definition of individualized treatment and the targeted use of new otoprotective drugs.
